# Menkes Disease Presenting with Epilepsia Partialis Continua

**DOI:** 10.1155/2014/525784

**Published:** 2014-11-23

**Authors:** Tamer Rizk, Adel Mahmoud, Tahani Jamali, Salah Al-Mubarak

**Affiliations:** ^1^Pediatric Neurology Department, Al-Takhassusi Hospital, HMG, P.O. Box 365814, Riyadh 11393, Saudi Arabia; ^2^Pediatric Neurology Department, National Neurosciences Institute, King Fahad Medical City, Riyadh, Saudi Arabia; ^3^Pediatric Neurology Division, University of Saskatchewan, Saskatoon, Canada

## Abstract

*Aim*. We aim to describe a female patient with Menkes disease who presented with epilepsia partialis continua. *Case Presentation*. Seventeen-months-old Saudi infant was presented with repetitive seizures and was diagnosed to have epilepsia partialis continua. *Discussion*. Menkes disease (OMIM: 309400) is considered a rare, X-linked recessive neurodegenerative disorder resulting from a mutation in the gene coding for the copper transporting ATPase (ATP7A). Affected individuals usually present with kinky hair, skeletal changes, prolonged jaundice, hypothermia, developmental regression, decreased tone, spasticity, weakness, and therapy resistant seizures. *Conclusion*. Raising awareness of abnormal presentation of this rare disease may help in the control of seizures through subcutaneous copper supplementation.

## 1. Introduction

Menkes disease (OMIM: 309400) is an X-linked recessive neurodegenerative disorder, resulting from a mutation in the gene coding for the copper transporting ATPase (ATP7A). Affected individuals usually present with kinky hair, skeletal changes, prolonged jaundice, hypothermia, developmental regression, decreased tone, spasticity, weakness, and therapy resistant seizures [1].

In 1895, Kojewnikoff described a unique type of prolonged seizure, which he named epilepsia partialis continua (EPC) [2].

Epilepsia partialis continua is a kind of simple partial seizure defined as spontaneous regular or irregular clonic muscular twitching affecting a limited part of the body, sometimes aggravated by action or sensory stimuli, occurring for a minimum of one hour, and recurring at intervals of no more than ten seconds [3, 4].

## 2. Case Description

We report a case of a 17-month-old girl who presented to our hospital with continuous right hand movements, regression of milestones, and repetitive episodes of hypothermia. Parents are second degree cousins. Her mother is a 23-year-old housewife. Her father is a 30-year-old soldier. Both parents finished high school education, There is no history of recurrent abortions. There is no history of similar cases. They are living in Assir region, Saudi Arabia, with fair socioeconomic status. They have a 4-year-old normal healthy boy.

During pregnancy, mother noticed decreased fetal movements: with no polyhydramnios, no oligohydramnios. She was born full term through spontaneous vertex vaginal delivery with normal birth weight and head circumference. The mother and the child were discharged home next day after delivery in good condition.

She had multiple hospital admissions before she presented to our hospital for abnormal continuous rhythmic hand movements.

At the age of 14 months, she started to develop sudden onset of continuous right hand movements, where they went to a local hospital and her EEG was abnormal and was given intravenous medications without benefit.

She was intubated in the Pediatric Intensive Care Unit (PICU), given intravenous infusion medications, and she stayed for 2 weeks. Then she was extubated and transferred to the ward. She was discharged home unable to sit or hold things with hands and unable to speak. She was put on topiramate, phenobarbitone, and clonazepam.

At home, her mother did not give the antiepileptic medications. Seizures recurred again with the same semiology plus right mouth twitches and occasional deviation of both eyes to the right side. The parents tried traditional treatment without benefit. They went back to local hospital and underwent the same course of treatment.

She was shifted to another hospital and was investigated with lab works, EEG, and brain MRI and the parents were informed of normal results.

After 2 months, she started to have decreased oral intake, increased sputum secretion, and bluish discoloration of lips and face with no abnormal movements.

She had an EEG that was abnormal. She was admitted to the PICU for 2 days. Her EEG was normalized and she stopped seizing. She was discharged home on the aforementioned three antiepileptic medications.

One month later, she started to have decreased feeding, perioral bluish discoloration, and unresponsiveness. She was admitted to the hospital: needing escalating doses of her medications. She became unable to follow objects with her eyes and her right hand movements were increasing in intensity during awake time. They were described as rhythmic, continuous, not stopping when held, and lasting for 2 weeks. They were not associated with bluish discoloration or mouth deviation.

The parents mentioned that she had constipation and episodes of hypothermia.

They mentioned that she was able to transfer objects at the age of 7 months, waved bye-bye at 9 months, sat with support at 10 months, and used to say mama, baba, nanna (milk), and dada (grandma).

She was continued on the same antiepileptic medications.

Examination when she presented to us revealed smooth straight black colored hair, right upper limb rhythmic, and continuous movements. Weight was 8 kg below 3rd centile, length was 77 cm on the 25th centile, and head circumference was 45 cm between the 10th and 25th centiles. She was not fixing and not following. She had preserved light reflex with bilateral equal reactive pupils. Fundoscopy was normal. There was no facial asymmetry, and gag reflex was preserved. She was responding to sounds. She was not able to move her right upper and lower limbs. There were no fasciculations, but decreased axial tone, with appendicular hypotonia on the right more than the left side.

Reflexes were brisk (+3) over all the limbs, but more easily elicited on the right side with normal sensation to touch and pin prick. Spine, skin, and other systemic examinations were normal.

Her investigations included tandem mass spectrometry, urine gas chromatograph, erythrocyte sedimentation rate, copper, zinc, copper, 24 h urine, ceruloplasmin, uric acid, very long chain fatty acids, C3,C4, serum ammonia, serum lactate, plasma amino acids, and cerebrospinal fluid (CSF) (glucose, protein, Herpes Simplex virus, Epstein Barr virus, Cytomegalovirus, amino acids, ammonia, lactate). They were all within normal.

Her electroencephalogram (EEG) showed excessive fast activity in the beta range over the posterior head regions and sharp waves at left temporoparietal region. The sharp waves were not time locked with the right hand rhythmic activity. Rare sharp transients and sharp waves were seen independently at right temporal at T8. There were prolonged episodes of the right hand rhythmic clonic activity that occurred episodically and lasted anywhere from 42 minutes to almost 1 hour. The patient had these events while awake as well as during sleep but with less intensity. There were no changes in her level of consciousness or her vital signs during these events. The events did not involve any other body parts apart from the right hand. Distraction of the patient's attention did not change movements.

**Figure 1:**
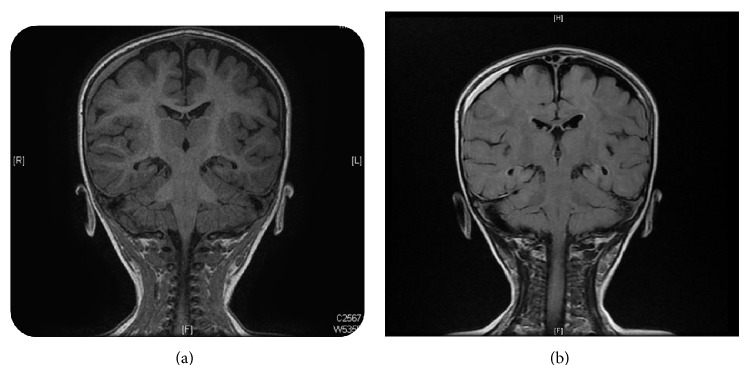
Brain magnetic resonance imaging (MRI) showed cortical and cerebellar atrophy, right-sided subdural collection noted over the right frontal and parietal convexity.

**Figure 2:**
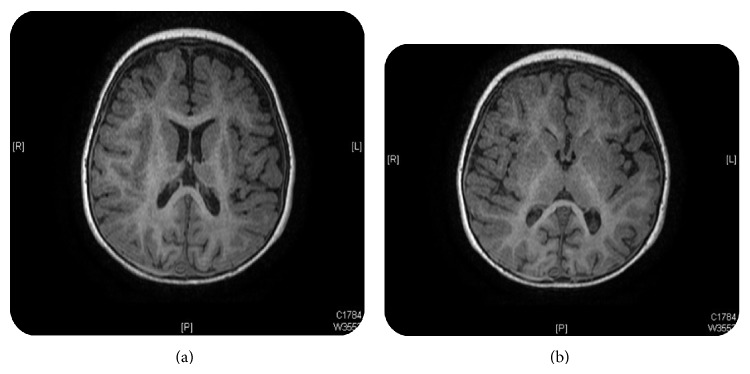
White matter T2 hyperintensity involving the centrum semiovale and the occipital lobes.

Brain magnetic resonance imaging (MRI) showed cortical and cerebellar atrophy, right-sided subdural collection noted over the right frontal and parietal convexity (Figures 1(a) and 1(b)); however, there was no associated compression effect noted over the underling cortical sulci. White matter T2 hyperintensity involving the centrum semiovale and the occipital lobes (Figures 2(a) and 2(b)). Evidence of right frontal cortically located lesion showing diffusion restriction indicating cytotoxic edema due to ischemia. Interval development of bilateral and symmetrical basal ganglia T2 hyperintensities affecting the putamina and thalami as well as the dentate nuclei.

Diffuse tensor imaging (DTI) showed reduction of the diffusion anisotropy noted in the left occipital cortical lesion with reduced white matter thickness of the sagittal stratum.

**Figure 3:**
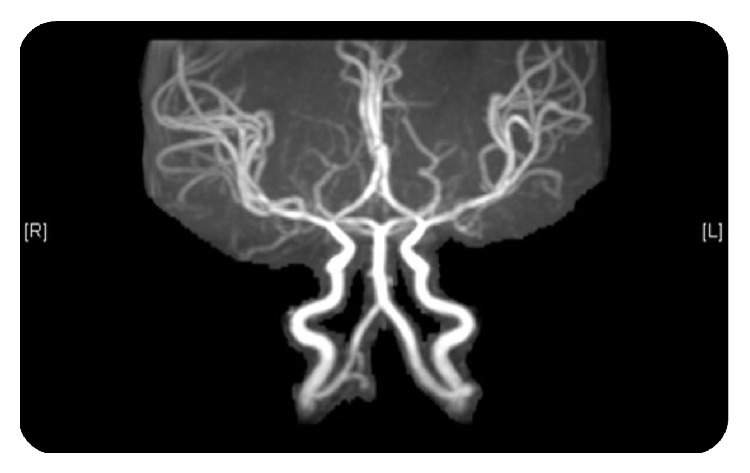
Magnetic resonance angiography (MRA) showed relatively tortuous but patent intracranial vessels, with appearance of “hair pin” sign.

Magnetic resonance angiography (MRA) showed relatively tortuous but patent intracranial vessels, with appearance of “hair pin” sign (Figure 3).

**Figure 4:**
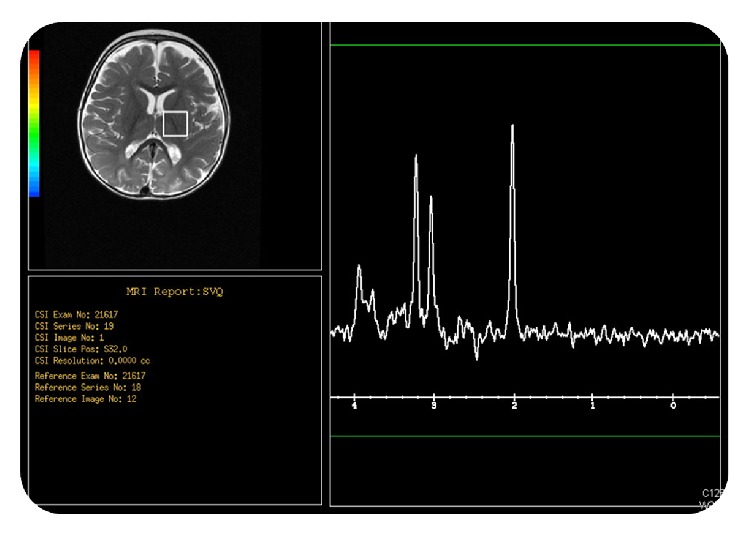
Magnetic resonance spectroscopy (MRS) obtained at the level of internal capsule demonstrated low choline peak.

**Figure 5:**
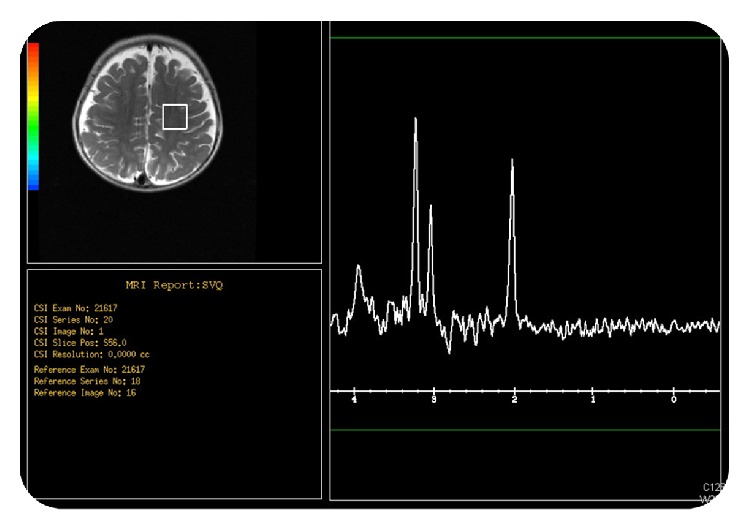
Magnetic resonance spectroscopy (MRS) obtained at the level of the parietal white matter demonstrated reduced N-acetyl aspartate peak at indicating neuronal loss.

Magnetic resonance spectroscopy (MRS) obtained at the level of internal capsule and at the parietal white matter demonstrated reduced N-acetyl aspartate peak at the parietal white matter indicating neuronal loss with low choline peak at the internal capsule (Figures 4 and 5).

Echocardiogram done on 10-04-2012 showed small ASD secundum.

Hair biopsy done on 10-04-2012 showed that most of the hair shafts examined under light microscopy were unremarkable. Few hair shafts show “Monilethrix abnormality.” One hair shaft shows fracture “Trichorrhexis Nodosa.”

*Genetic Study*. Revealed the presence of a heterozygous mutation, C.1138G>A (p.Val380Met) in the ATP7A gene.

## 3. Discussion

Menkes disease is a rare X-linked recessive neurodegenerative disorder, nearly exclusively affecting males. It results from mutation in the gene coding for the copper transporting ATPase (ATP7A) [5].

Affected infants are healthy at birth and have early normal development for the first 2–4 months and then recognition of seizures and hypotonia and failure to thrive begin and they often die by the age of 3 years [6].

They have characteristic hair that is short, scanty, and hypopigmented [6].

The hair is even shorter on the sides and the back of the head than on the top. The twisted strands may be reminiscent of those in steel wool cleaning pads. The eyebrows usually share the unusual appearance. Light microscopy of patient hair illustrates pathognomonic pili torti (i.e., 180° twisting of hair shaft) and often other abnormalities including trichoclasis (i.e., transverse fracture of hair shaft) and trichoptilosis (i.e., longitudinal splitting of shaft). Hair may demonstrate unusual colors, such as white, silver, or grey; however, in some individuals with Menkes disease, the hair is pigmented normally.

The typical patients have jowly faces, with sagging cheeks and ears that often appear large. They have high-arched palates, with delayed teeth eruption [7].

Profound truncal hypotonia is invariably present. This may be accompanied with increased appendicular tone. Deep tendon reflexes are often hyperactive. The suck and cry are usually strong [8]. Visual fixation and tracking commonly are impaired [9].

Epilepsy is found to be a major feature where seizures are usually of focal pattern which may progress to epileptic spasms in the early stages and as tonic seizures and myoclonic jerks in the late stages [10].

West syndrome as an epileptic presentation in Menkes' was reported in two cases [11].

Neuroimaging studies show atrophy of grey matter, ventriculomegaly, and tortuous intracranial vasculature with white matter signal changes consistent with loss of myelin and axons [12].

Cerebrospinal fluid is characterized by the presence of lactic acidosis suggesting widespread disturbance in oxidative metabolism [13].

Molecular consequences of the pathogenic ATP7A gene mutation lead to impairment in copper transport, which in turn causes deficiencies of key copper containing enzymes (dopamine *β* hydroxylase and cytochrome c oxidase). Microarray studies suggest widespread effects in dysregulation of genes involved in cellular responses to oxidative stress, ribosomal translation, signal transduction, mitochondrial function, and immune responses. Impairment of copper mediated NMDA receptor function further enhances neuronal excitability and excitotoxic neuronal injury, setting up a cascade that creates conditions for epileptogenesis to follow [13].

Early diagnosis and start of copper supplementation have been shown to be beneficial and improve brain electrical activity and decrease seizure occurrence in classical Menkes disease irrespective of the precise molecular defect [13].

Children with this X-chromosomal, recessive disorder almost always suffer from epilepsy, often presenting with infantile spasms resistant to treatment [14].

The characteristic hair abnormalities point to the diagnosis, which can be confirmed by low serum copper and ceruloplasmin. Administration of subcutaneous copper histidinate may stop seizures and improve development [15].

There are variants of Menkes disease worth considering in differential diagnosis.

Occipital horn syndrome refers to the pathognomonic wedge-shaped calcifications that form bilaterally within the trapezius and sternocleidomastoid muscle tendons at their attachment to the occiput in affected individuals [16].

Patients have lax skin and joints, bladder diverticula, inguinal hernia, vascular tortuosity, and normal or slightly subnormal intelligence [17].

Menkes and occipital horn syndrome gene mutation analysis from genomic DNA has been conducted.

This generated important information about the molecular correlates of certain clinical and biochemical phenotypes and about functional aspects of this copper-ATPase [18].

Female patients with Menkes disease have been reported, in whom chromosome rearrangement, XO/XX mosaicism, or unfavorable lyonization was responsible for expression of the full phenotype [19].

Epilepsia partialis continua is a subtype of simple partial status epilepticus characterized by continuous, involuntary focal muscle jerking of cortical origin occurring at least every 10 seconds for at least 1 hour and not impairing awareness.

Epilepsia partialis continua is a rare condition with a wide range of underlying etiologies; in children, the most common cause is Rasmussen syndrome, and in adults, the most common causes are cerebrovascular disease and neoplasm.

Epilepsia partialis continua is typically resistant to medications, and whenever possible, treatment should focus on the underlying cause [20].

To the best of our knowledge, and a review of the PubMed data base, a girl with Menkes disease with the above mentioned mutation presenting with epilepsia partialis continua was never reported before.

## 4. Conclusion

In our case the presentation of epilepsia partialis continua can be due to lesions caused by heterozygous mutation, C.1138G>A (p.Val380Met) in the ATP7A gene of Menkes disease.
